# Morphological and phylogenetic analyses of an *Elongisporangium* species and two *Globisporangium* species (Peronosporales, Oomycota) from China

**DOI:** 10.3389/fcimb.2026.1832868

**Published:** 2026-06-11

**Authors:** Yuanyuan Chen, Yifan Xing, Jian Yu, Jinfeng Peng, Jiajia Chen

**Affiliations:** 1 College of Forestry, Henan Agricultural University, Zhengzhou, China; 2 College of Landscape Architecture, Jiangsu Vocational College of Agriculture and Forestry, Zhenjiang, China; 3 Zhejiang Key Laboratory of Soil Remediation and Quality Improvement, Zhejiang A&F University, Hangzhou, China

**Keywords:** diversity, molecular phylogeny, morphology, oomycetes, *Pythium* sensu lato

## Abstract

**Introduction:**

The species diversity and geographical distribution of Pythium sensu lato (s.l.) in China remain to be further supplemented and improved. In this study, three species formerly classified under Pythium s.l., Elongisporangium senticosum, Globisporangium monoclinum, G. oryzicola were new records from China.

**Methods:**

Illustrated descriptions of the three new Chinese records are given based on their morphological observations and phylogenetic analysis inferred from the internal transcribed spacer region of the ribosomal RNA gene and mitochondrial cytochrome c oxidase subunit I gene.

**Results:**

E. senticosum was found in Hainan and Guangdong Provinces, and it is characterized by having ovoid to ellipsoidal sporangia with conspicuous apical papilla, ornamented oogonia with acute spines, mostly monoclinous antheridia, and aplerotic oospores; G. monoclinum was collected from Xinjiang Uygur Autonomous Region, and it is distinguished by presence of hyphal swellings, absence of sporangia, smooth oogonia, mostly monoclinous antheridia, and subcircular, subclavate or fist-shaped antheridial cells; G. oryzicola was found in Jiangsu province, and it is characterized by having rarely presence of hyphal swellings, absence of sporangia, monoclinous or diclinous, sometimes hypogynous antheridia, and thin-walled oospores (0.8–1.1 µm).

**Discussion:**

This study provides valuable insights into the distribution of oomycetes in China.

## 1 Introduction

The genus *Pythium* sensu lato (s.l.) Pringsheim was established in 1858 with the type species *P. monospermum* Pringsh (Pringsheim, 1858) and characterized by the production of zoospores in a vesicle that develops at the tip of a discharge tube derived from a sporangium (van der Plaäts-Niterink, 1981). Species of *Pythium* s.l. exhibit diverse lifestyles, including saprophytic, parasitic, and facultatively parasitic modes, and are widely distributed in terrestrial and aquatic habitats (van der Plaäts-Niterink, 1981; Uzuhashi et al., 2015). More than 170 species of *Pythium* s.l. have been described worldwide (Uzuhashi et al., 2015; Ueta and Tojo, 2016; Salmaninezhad and Mostowfizadeh-Ghalamfarsa, 2019; Chen et al., 2022; Eggertson et al., 2023; Liu et al., 2023; Ahadi et al., 2024; Joseph et al., 2024; Kikuchia et al., 2024; Salmaninezhad et al., 2024; Otsubo et al., 2025), which include many important plant pathogens, causing damping-off, stunting, blight, seedling rot, stem rot, root rot, flower rot, and postharvest rot of fruits, vegetables, and seeds (Ho and Abd-elsalam, 2020; Bickel and Koehler, 2021). Some species induce host resistance and have potential for use as biocontrol agents (Ali-Shtayeh and Saleh, 1999), while others are important pathogens of animals (Shen et al., 2019).

*Pythium* s.l. was initially split into 11 clades (A–K) proposed by Lévesque and de Cock (2004). Following a series of taxonomic revisions, recombinations, and updates, the broadly defined genus *Pythium* s.l. currently comprises *Pythium* sensu stricto (s.s.), *Globisporangium*, *Elongisporangium*, *Phytopythium*, and *Pilasporangium* (Uzuhashi et al., 2010; Nguyen et al., 2022). *Globisporangium* (clades E–G, I, J) is typified with *G. paroecandrum* (Drechsler) Uzuhashi, Tojo & Kakish., and is characterized by presence of globose hyphal swellings or sporangia (Uzuhashi et al., 2015). Presently, 108 species are accepted under this genus (Nguyen et al., 2022). *Elongisporangium* (clade H), typified by *E. anandrum* (Drechsler) Uzuhasi, Tojo & Kakish., includes species with clavate or elongated sporangia. Eight species of *Elongisporangium* are recognized worldwide (Otsubo et al., 2025).

During studies on the diversity of *Pythium* s.l. species in China, three new Chinese records were identified based on morphological characters and molecular phylogenetic analyses of the internal transcribed spacer (ITS) and cytochrome c oxidase subunit I (*Cox1*) sequence data. These three species are described and illustrated in this study. Moreover, comparisons of these three species and their morphologically and phylogenetically related species are also provided.

## 2 Materials and methods

### 2.1 Isolation

Soil cores (4 cm in diameter, 10 cm in depth) were collected in each site. Soil samples were placed in an ice-cooled container, transported to the laboratory, and stored at 4°C until processing. For each soil sample, 0.10–0.25-mg unsieved soil particles were selected with sterilized forceps and then placed on selective V8 juice agar (V8A) medium containing rifampicin (50 mg/l), phenamacril (5 mg/l), ampicillin (50 mg/l), and pentachloronitrobenzene (50 mg/l) and incubated at 25 °C for 2–3 days. When mycelial growth was observed, a small piece of medium with mycelia at the edge of a colony was cut and transferred onto the new V8A medium plates. The purification process was repeated three to five times.

### 2.2 Morphology and growth rate

The studied cultures and the dried specimens were deposited in the herbarium of the College of Landscape Architecture, Jiangsu Polytechnic College of Agriculture and Forestry, Zhenjiang (JAFLA). The purified isolates were grown on V8A for morphological studies. Pure isolates grown on V8A for 5–7 days were cut into 5 mm × 5 mm plugs, transferred to glass Petri dishes containing sterile distilled water, and supplemented with sterile soil extract per dish. The dishes were incubated at 25 °C under light for 48 h to induce formation of sporangia. The soil extract was prepared according to De Cock and Lévesque (2004). A total of 50 measurements were taken for each morphological feature, such as sporangia, oogonia, and oospores. The cardinal temperatures were examined on potato carrot agar (PCA) according to the method of van der Plaäts-Niterink (1981), and growth rates were measured at 24-h incubation. Each isolate was incubated at 5 °C–40 °C with intervals of 5 °C on PCA media. When no growth was observed, the intervals were reduced from 5 to 2 or 1 °C and the culture was returned to room temperature to check the revival of the growth.

### 2.3 DNA extraction and sequencing

A cetyl trimethylammonium bromide rapid plant genome extraction kit (Demeter Biotechnologies Co., Ltd., Beijing) was used to extract total genomic DNA from purified isolates, and polymerase chain reaction (PCR) was performed according to Chen et al. (2017).

The ITS region was amplified with the primers ITS4 (TCCTCCGCTTATTGATATGC) and ITS5 (GGAAGTAAAAGTCGTAACAAGG) (White et al., 1990). The Cox1 gene was amplified with the primers OomCoxI-Levlo (CYTCHGGRTGWCCRAAAAACCAAA) and OomCoxI-Levup (TCAWCWMGATGGCTTTTTTCAAC) (Robideau et al., 2011). The PCR procedure for ITS was as follows: initial denaturation at 95 °C for 3 min, followed by 35 cycles at 94 °C for 40 s, 54 °C for 45 s, 72 °C for 1 min, and a final extension of 72 °C for 10 min. The PCR procedure for Cox1 was as follows: initial denaturation at 94 °C for 2–5 min, followed by 35 cycles at 94 °C for 30 s, 52 °C for 30 s, 72 °C for 1–2 min, and a final extension of 72 °C for 5–10 min (Blair et al., 2008). The PCR products were purified and sequenced in GenScript company (Nanjing, China).

### 2.4 Phylogenetic analysis

Sequences generated in this study were aligned with additional sequences downloaded from GenBank (**Table 1**) using ClustalX (Thompson et al., 1997) and manually adjusted in BioEdit (Hall, 1999). Phylogenetic analysis was performed following the methods of Cui et al. (2019). Maximum parsimony (MP) analysis was applied to the combined datasets of ITS and Cox1 sequences. Two isolates of *Saprolegnia parasitica* Coker, CBS 113187 and CBS 540.67, were used as outgroups. The MP tree construction procedure was performed in PAUP* version 4.0b10 (Swofford, 2002). All characters were equally weighted, and gaps were treated as missing data. Trees were inferred using the heuristic search option with TBR branch swapping and 1,000 random sequence additions. Max-trees were set to 5,000, branches of zero length were collapsed, and all parsimonious trees were saved. Clade robustness was assessed using a bootstrap (BT) analysis with 1,000 replicates (Felsenstein, 1985). Descriptive tree statistics tree length (TL), consistency index (CI), retention index (RI), rescaled consistency index (RC), and homoplasy index (HI) were calculated for each maximum parsimonious tree (MPT) generated. Phylogenetic trees were visualized using TreeView (Page, 1996).

**Table 1:** A list of species, cultures, and GenBank accession numbers of sequences used in this study.

Species name	Sample no.	locality	GenBank accession no.	
			ITS	*Cox1*
*Elongisporangium anandrum*	CBS 285.31	–	HQ643435	HQ708482
*E. dimorphum*	CBS 406.72^T^	USA	HQ643525	HQ708571
*E. disparispinum*	NBRC 115123^T^	Japan	LC805899	LC805908
*E. helicandrum*	CBS 393.54^T^	USA	HQ643548	HQ708592
*E. prolatum*	CBS 845.68^T^	USA	HQ643754	HQ708795
*E. senticosum*	CBS 122490^T^	Japan	AB362166	HQ708814
*E. senticosum*	Chen 1002	China	PX230419^*^	PX214364^*^
*E. senticosum*	Chen 1114	China	PX230420^*^	PX214365^*^
*E. undulatum*	CBS 157.69	USA	HQ643946	HQ708987
*E. verrucosum*	NBRC 115636^T^	Japan	LC805897	LC805905
*Globisporangium abappressorium*	CBS 110198^T^	USA	HQ643408	HQ708455
*G. capense*	CBS 149752^T^	Australia	OL342598	OL331986
*G. commune*	CBS 149753^T^	Australia	OL952618	OL860920
*G. cylindrosporum*	CBS 218.94^T^	Germany	HQ643516	HQ708562
*G. debaryanum*	CBS 752.96	United Kingdom	HQ643519	HQ708565
*G. emineosum*	DAOM BR836	–	GQ244428	GQ244424
*G. huanghuaiense*	Chen 94^T^	China	MF984118	MF984155
*G. irregulare*	CBS 250.28	Netherlands	HQ643596	HQ708640
*G. iwayamai*	CBS 156.64	Australia	HQ643669	HQ708713
*G. kunmingense*	CBS 550.88^T^	China	HQ643672	HQ708716
*G. lucens*	CBS 113342	Canada	HQ643681	HQ708725
*G. macrosporum*	CBS 574.80	Netherlands	HQ643684	HQ708728
*G. mahabadense*	IRAN 4986C^T^	Iran	PQ037626	PQ031213
*G. mamillatum*	CBS 251.28	Netherlands	HQ643687	HQ708731
*G. minor*	CBS 226.88^T^	United Kingdom	HQ643696	HQ708740
*G. monoclinum*	IRAN 2421 C^T^	Iran	MH203014	MG182702
*G. monoclinum*	Chen 1725	China	PX230421^*^	PX214366^*^
*G. oryzicola*	CBS 142206^T^	Japan	LC169733	LC169739
*G. oryzicola*	Chen 509	China	PX230422^*^	PX214367^*^
*G. oryzicola*	Chen 510	China	PX230423^*^	PX214368^*^
*G. oryzicola*	Chen 511	China	PX230424^*^	PX214369^*^
*G. paroecandrum*	CBS 157.64	Australia	HQ643731	HQ708772
*G. pengfuense*	Chen 93^T^	China	MF984129	MF984166
*G. rostratum*	CBS 53374	Netherlands	HQ643767	HQ708808
*G. spiculum*	CBS 122645^T^	France	HQ643790	HQ708831
*G. spinosum*	CBS 27667	Netherlands	HQ643792	HQ708833
*G. splendens*	CBS 462.48	USA	HQ643795	HQ708836
*G. sylvaticum*	CBS 453.67^T^	USA	HQ643845	HQ708886
*G. tabrizense*	IRAN 4985C^T^	Iran	PQ037624	PQ031210
*G. terrestris*	CBS 112352^T^	France	HQ643857	HQ708898
*G. ultimum*	CBS 398.51^T^	Netherlands	HQ643865	HQ708906
*G. violae*	CBS 159.64	Australia	HQ643958	HQ708999
*Phytopythium boreale*	CBS 551.88	China	AB725879	AB690647
*Pp. vexans*	CBS 119.80	Iran	HQ643400	HQ708447
*Pythium adhaerens*	CBS 52074	Netherlands	HQ643415	HQ708462
*P. agreste*	HMAS 243737^T^	China	HE862395	HE862399
*P. brachiatum*	UZ00735	Japan	KJ995582	KJ995592
*P. grandisporangium*	CBS 286.79^T^	USA	AY598692	HQ708590
*P. inflatum*	CBS16868	USA	HQ643566	HQ708610
*P. insidiosum*	CBS 574.85^T^	Costa Rica	HQ643570	HQ708614
*P. junctum*	UZ00732^T^	Japan	KJ995576	KJ995595
*P. monospermum*	CBS 158.73	United Kingdom	HQ643697	HQ708741
*P. oligandrum*	CBS 382.34	United Kingdom	AY598618	HQ708759
*P. periplocum*	CBS 289.31^T^	USA	AY598670	HQ708784
*P. plurisporium*	CBS 100530^T^	USA	HQ643749	HQ708790
*P. sukuiense*	CBS 110030^T^	China	HQ643836	HQ708877
*Saprolegnia parasitica*	CBS 113187	Russia	HQ644005	HQ709046
*S. parasitica*	CBS 540.67	United Kingdom	HQ644000	HQ709041

* New sequences determined in the present study. ^T^Ex-type strains.

MrModeltest 2.3 (Nylander, 2004) was used to determine the best-fit evolution model for Bayesian Inference (BI). BI of the dataset was calculated with MrBayes 3.1.2 (Ronquist and Huelsenbeck, 2003) with a general time reversible (GTR) model of DNA substitution and an inverse gamma distribution rate variation across sites. Four Markov chains were run for two runs from random starting trees for 2 million generations of the two combined datasets, and trees were sampled every 100 generations. The burn-in was set to discard the first 25% of the trees. A majority rule consensus tree of all remaining trees was calculated. Branches that received bootstrap support for MP and Bayesian posterior probabilities (BPP) greater than or equal to 75% (MP) and 0.90 (BPP) respectively were considered as significantly supported.

## 3 Results

### 3.1 Molecular phylogeny

Phylogenetic analyses of ITS or *Cox1* sequences of six isolates recovered from soil (those are identified here as *E. senticosum*, *G. monoclinum*, and *G. oryzicola*) showed that the best matches of six isolates were with the species of *Elongisporangium* and *Globisporangium*, respectively.

The combined ITS+*Cox1* dataset of *Pythium* s.l. species included sequences from 58 isolates representing 52 taxa. The dataset had an aligned length of 1,736 characters, of which 692 characters are constant, 144 are variable and parsimony-uninformative, and 900 are parsimony-informative. Maximum parsimony analysis yielded 22 equally parsimonious trees (TL = 4532, CI = 0.457, RI = 0.749, RC = 0.342, HI = 0.543). The best model for the combined ITS+*Cox1* sequences dataset estimated and applied in the Bayesian analysis: GTR+I+G, lset nst = 6, rates = invgamma; prset statefreqpr = dirichlet (1,1,1,1). Bayesian analysis resulted in the same topology with an average standard deviation of split frequencies = 0.009166.

**Figure 1:**
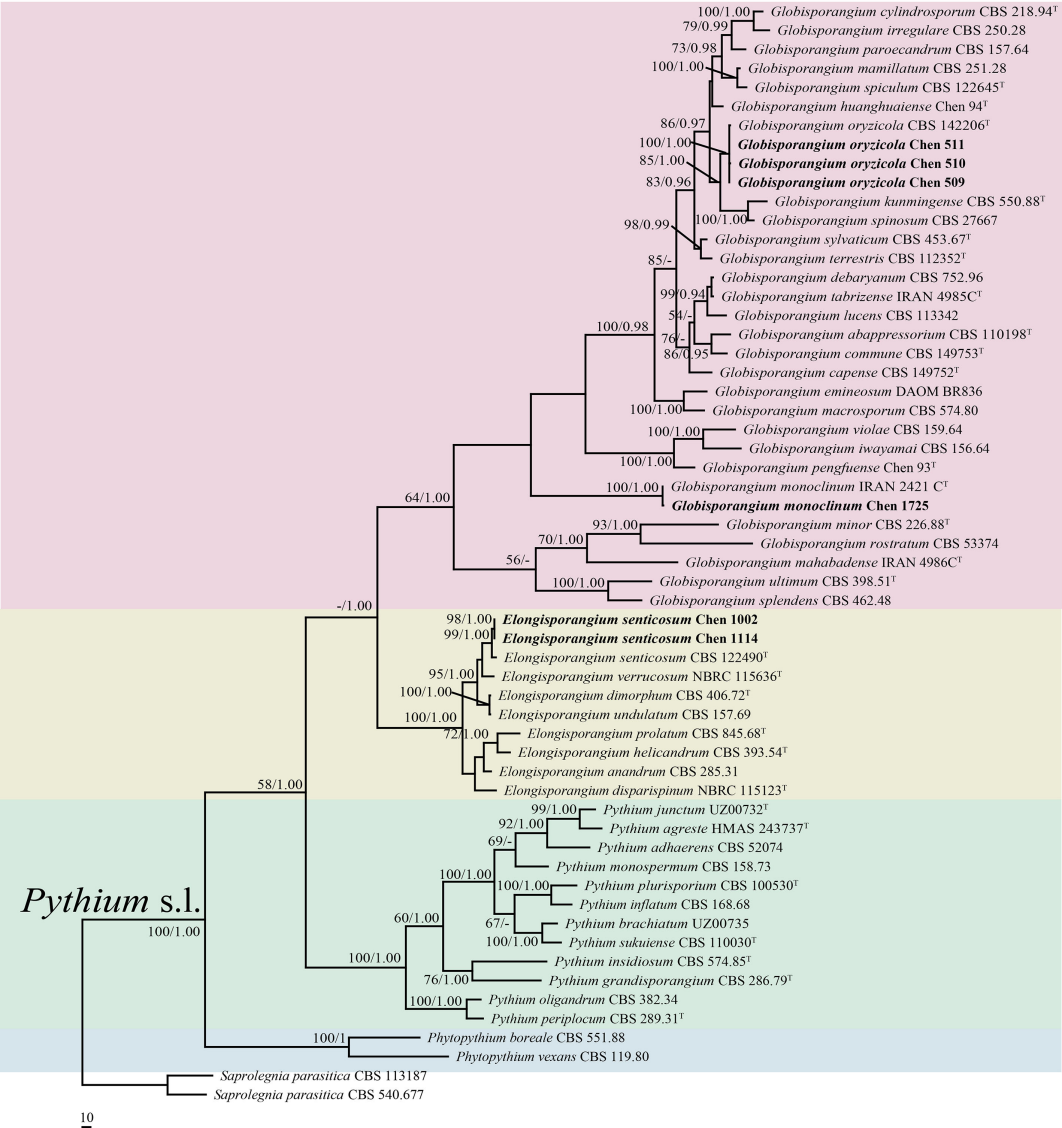
Phylogeny of *Elongisporangium* spp., *Globisporangium* spp., and related species generated by maximum parsimony based on ITS+*Cox1* sequences. Branches are labeled with parsimony bootstrap proportions (before slanting line) high than 50% and Bayesian posterior probabilities (after slanting line) more than 0.90.

The resulting phylogenetic tree was overall consistent with the one reported by Nguyen et al. (2022). Our three collections were identified as three distinct species, each clustering with *E. senticosum*, *G. monoclinum*, and *G. oryzicola*, all supported by high bootstrap values (**Figure 1**).

### 3.2 Taxonomy

**Figure 2:**
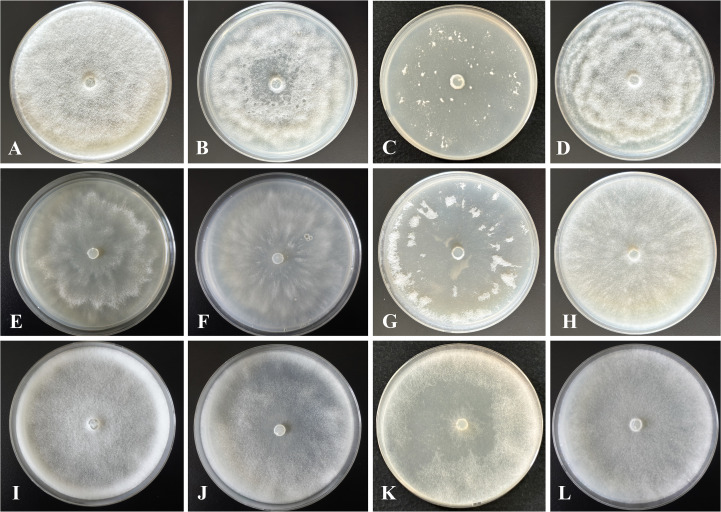
Colony patterns of *Elongisporangium* species and *Globisporangium* species on CMA, PCA, V8A, and PDA, respectively. **(A–D)** *E. senticosum* (Chen 1002). **(E–H)** *G. monoclinum* (Chen 1725). **(I–L)** *G. oryzicola* (Chen 509).

**Figure 3:**
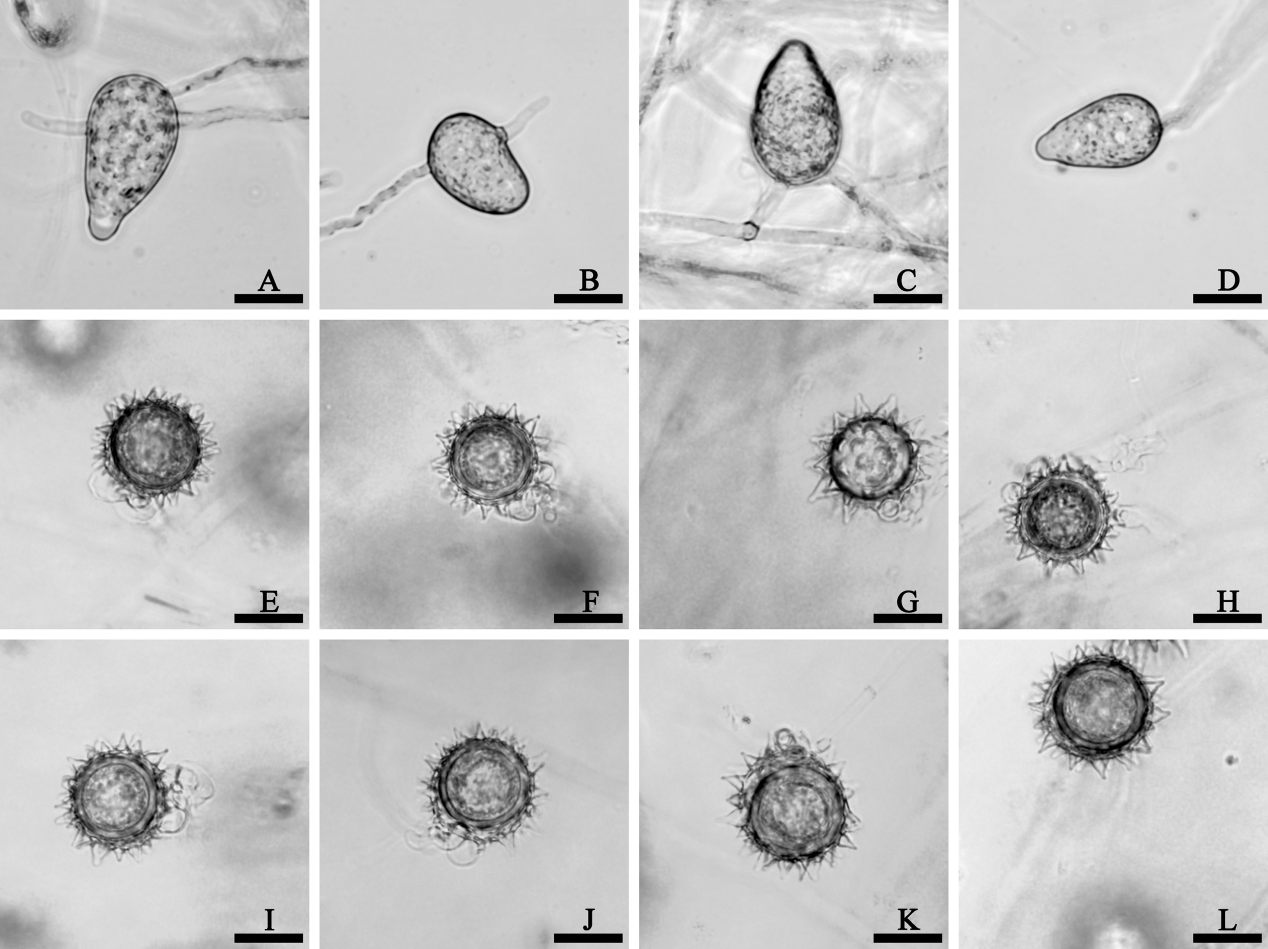
Asexual and sexual reproductive bodies of *Elongisporangium senticosum* (Chen 1002). **(A)** Ovoid sporangium with papilla. **(B)** Intercalary sporangium. **(C, D)** Terminal sporangium with semipapilla. **(E–G)** Ornamented oogonia with monoclinous antheridia. **(H)** Clustering of several antheridial cells around the oogonium. **(I–J)** Two monoclinous antheridia. **(K)** Oogonium with a projection. **(L)** aplerotic oospore. Scale bars A–L=20 μm.

*Elongisporangium senticosum* (Senda & Kageyama) H.D.T. Nguyen & C.F.J. Spies, Mycologia 114(3): 508 (2022) (**Figures 2**, **3**).

MycoBank number: MB 840708

Colonies submerged, with cottony pattern on corn meal agar (CMA), cottony rosette pattern on PCA, without a special pattern on V8A, and cottony rosette pattern on potato dextrose agar (PDA) (**Figures 2A–D**). Average growth rates 4 mm day−1 at 5 °C, 12 mm day−1 at 10 °C, 16 mm day−1 at 15 °C, 21 mm day−1 at 20 °C, 25 mm day−1 at 25 °C, 5 mm day−1 at 30 °C. Cardinal temperatures: minimum 4 °C, optimum 25 °C, maximum 35 °C. Main hyphae hyaline, aseptate, up to 6.0 µm wide. Hyphal swellings absent. Sporangia ovoid to ellipsoidal with conspicuous apical papilla, terminal or intercalary, 29.5–54.5 × 16.5–33.5 (mean 43.5 × 25.5) μm (**Figures 3A–D**). Homothallic; oogonia globose, ornamented, mainly terminal, rarely intercalary, 23–32 µm (mean 26.5 µm) in diameter. Antheridia mostly monoclinous, rarely diclinous, one to three per oogonium (**Figures 3E–L**); antheridial stalks unbranched; antheridial cells elongated along the oogonial stalk, semicircle, subclavate or fist-shaped. Oospores aplerotic, globose, 19.5–26.5 μm (mean 23.5 µm) in diameter, hyaline. Oospore wall 1.0–2.5 µm (mean 1.9 µm) thick.

Material examined.—CHINA. Hainan Province: Qiongzhong Li and Miao Autonomous County, Limushan Nature Reserve, 109.76°E, 19.22°N, from the soil of *Dacrydium pectinatum*, 11 November 2020, Chen 1002 (JAFLA 1247); Guangdong Province: Yunfu, Yunan County, Tongledashan Nature Reserve, 111.41°E, 23.22°N, from the soil of *Pinus massoniana*, 24 December 2020, Chen 1114 (JAFLA 1570). The isolate was also maintained in freeze-dried cultures.

**Figure 4:**
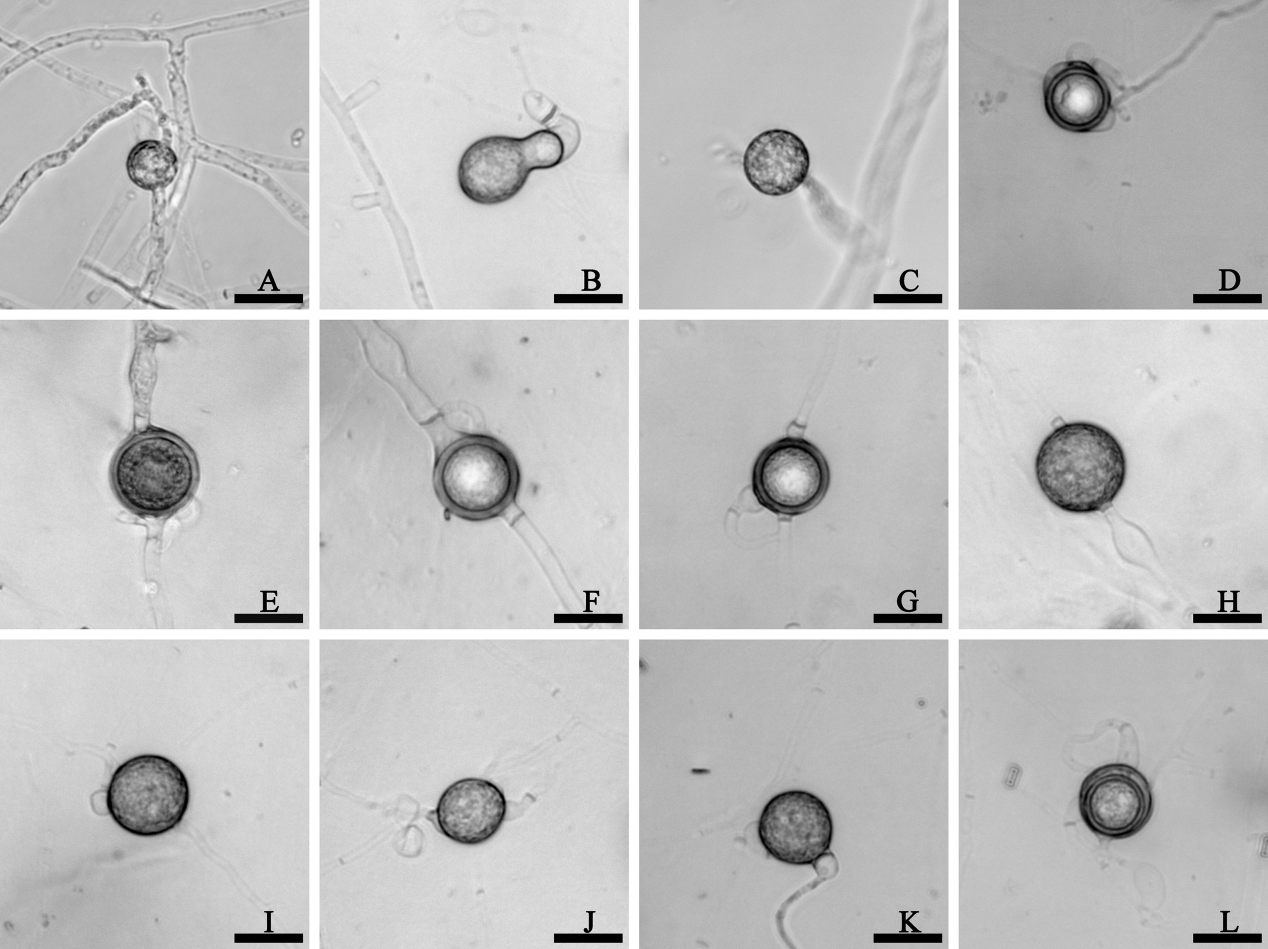
Asexual and sexual reproductive bodies of *Globisporangium monoclinum* (Chen 1725). **(A)** Intercalary hyphal swelling. **(B)** Filamentous inflated hyphal swelling. **(C)** Globose hyphal swelling. **(D)** Clustering of several antheridial cells around the oogonium. **(E–G)** Intercalary oogonia with monoclinous antheridia. **(H)** Hypogynous antheridium. **(I–K)** Plerotic oospore. **(L)** Nearly plerotic oospore and two antheridia. Scale bars A–L=20 μm.

*Globisporangium monoclinum* (Abrinbana, Abdollahz. & Badali) H.D.T. Nguyen & C.F.J. Spies, Mycologia 114(3): 508 (2022) (**Figures 2**, **4**).

MycoBank number: MB 840733

Colonies submerged, with mixed rosette and chrysanthemum patterns on CMA, chrysanthemum pattern on PCA, cottony radiate pattern on V8A, and cottony pattern on PDA (**Figures 2E–H**). Average growth rates 2 mm day−1 at 5 °C, 5 mm day−1 at 10 °C, 10 mm day−1 at 15 °C, 13 mm day−1 at 20 °C, 16 mm day−1 at 25 °C, 12 mm day−1 at 30 °C, 6 mm day−1 at 35 °C. Cardinal temperatures: minimum 4 °C, optimum 25 °C, maximum 38 °C. Main hyphae hyaline, branched, aseptate, up to 6.0 µm wide. Hyphal swellings globose to sub-globose, sometimes filamentous inflated, terminal or intercalary, 14.6–26.4 (mean 22.9) μm in diameter (**Figures 4A–C**). Sporangia and zoospores not observed. Homothallic; oogonia globose, smooth, mostly intercalary, occasionally terminal, 18.8–25.8 µm (mean 21.5 µm) in diameter. Antheridia mostly monoclinous, sometimes hypogynous or diclinous, one to two per oogonium (**Figures 4D–L**); antheridial stalks unbranched; antheridial cells elongated along the oogonial stalk, subcircular, subclavate or fist-shaped. Oospores plerotic or aplerotic, globose, 15.3–22.6 μm (mean 18.7 µm) in diameter, hyaline. Oospore wall 1–2 µm (mean 1.6 µm) thick.

Material examined.—CHINA. Xinjiang Uygur Autonomous Region: Hetian, Pishan County, Muji Service Area, 78.49°E, 37.44°N, from the soil of the desert, 27 May 2023, Chen 1725 (JAFLA 3739). The isolate was also maintained in freeze-dried cultures.

**Figure 5:**
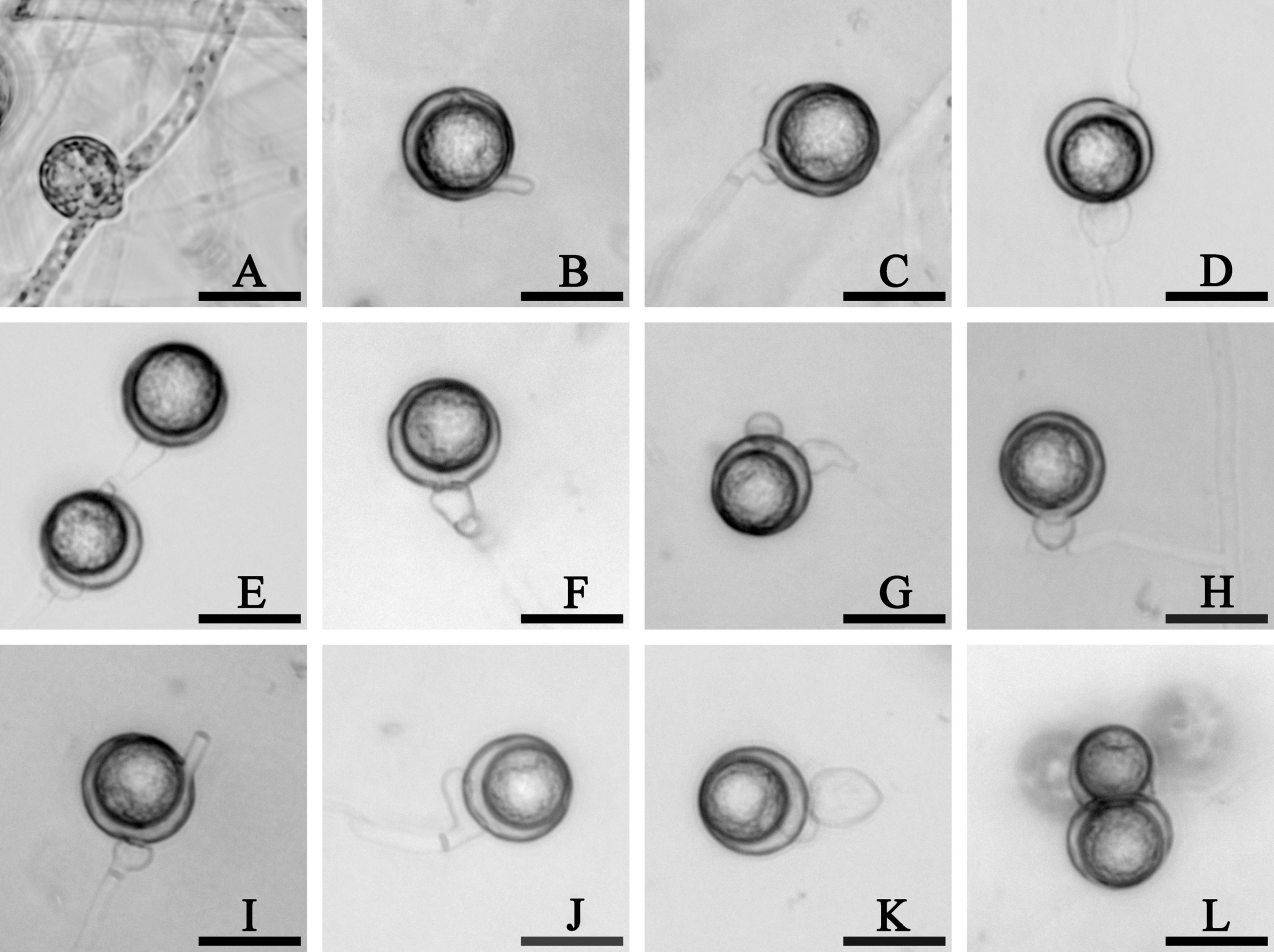
Asexual and sexual reproductive bodies of *Globisporangium oryzicola* (Chen 509). **(A)** Intercalary hyphal swelling. **(B)** Oogonium with a projections. **(C, D)** Monoclinous antheridia. **(E)** Chain oogonium. **(F)** Globose oogonia oogonium with one antheridium. **(G)** Two antheridia. **(H)** Diclinous antheridium. **(I)** Hypogynous antheridium. **(J)** Terminal oogonium. **(K)** Oval antheridial cell. **(L)** Aplerotic oospores. Scale bars A–L=20 μm.

*Globisporangium oryzicola* Uzuhashi & Tojo, Antonie van Leeuwenhoek 110(4): 550 (2017) (**Figures 2**, **5**).

MycoBank number: MB 817839

Colonies submerged, with cottony patterns on CMA, PCA, V8A, and PDA (**Figures 2I–L**). Average growth rates 6 mm day−1 at 10 °C, 10 mm day−1 at 15 °C, 13 mm day−1 at 20 °C, 25 mm day−1 at 25 °C, 20 mm day−1 at 30 °C. Cardinal temperatures: minimum 5 °C, optimum 28 °C, maximum 35 °C. Main hyphae hyaline, aseptate, up to 7.0 µm wide. Main hyphae hyaline, branched, aseptate, up to 6.0 µm wide. Hyphal swellings rarely observed, globose to sub-globose, mainly intercalary, occasionally terminal, 14.5–21.5 (mean 17.9) μm in diameter (**Figure 5A**). Sporangia and zoospores not observed. Homothallic; oogonia globose, smooth or with a projection, terminal or intercalary, 16.5–23.5 (mean 20.4) in diameter. Antheridia monoclinous or diclinous, sometimes hypogynous, one to two per oogonium (**Figures 5B–L**); antheridial stalks unbranched; antheridial cells elongated along the oogonial stalk, various in shapes, subcircular, sub-globose, fist-shaped, subclavate or oval. Oospores mostly aplerotic, occasionally plerotic, globose, 14.7–19.3 μm (mean 16.2 µm) in diameter, hyaline. Oospore wall 0.8–1.1 µm (mean 1.0 µm) thick.

Material examined.—CHINA. Jiangsu Province: Nanjing, Jiangning District, Tuqiao Village, 119.07°E, 31.94°N, from the soil of *Oryza sativa*, 10 May 2019, Chen 509–511 (JAFLA 314, 316, 318). The isolate was also maintained in freeze-dried cultures.

## 4 Discussion

To date, 77 species of *Pythium* s.l. have been reported from China (Long et al., 2012, 2014; Ho, 2013; Chen et al., 2020, 2022, 2025; Zheng et al., 2026). As part of an ongoing study of the diversity of *Pythium* s.l. species in China, the cultures (Chen 509-511, 1002, 1114, and 1725) were isolated and identified as three new Chinese records, *E. senticosum*, *G. monoclinum*, and *G. oryzicola* with the aid of morphological characters and phylogenetic analyses based on ITS and *Cox1* gene sequences combined. Detailed morphological comparisons between these new Chinese records and their closely related taxa are provided in **Tables 2**–**4**.

**Table 2:** Morphological description of *Elongisporangium senticosum* and the most closely related species.

Character	*E. senticosum* (Chen 1002)	*E. senticosum* (CBS 122490, ex-type strain)	*E. dimorphum*	*E. disparispinum*	*E. verrucosum*
Width of hyphae (μm)	Up to 6	Up to 8	Up to 10	Up to 6	Up to 6.9
Sporangia	Ovoid to ellipsoidal, papillate, terminal or intercalary	Ovoid to ellipsoidal, papillate, terminal	Ovoid, elongated, ellipsoidal or clavate, terminal	Globose to ovoid, papillate at maturity, terminal	Ovoid, elongated, often irregularly shaped, papillate, terminal
Chlamydospores	Absent	Absent	Globose, av. 50	Absent	Globose, av. 33.1
Oogonia (μm)	Ornamented with acute conical spines, 23–32 (av. 26.5), mainly terminal, rarely intercalary	Ornamented with acute conical spines, 19–32 (av. 26.4), mainly terminal, occasionally intercalary	Ornamented with bluntly conical projections, terminal	Ornamented with short acute conical spines, 19.8–28 (av. 22.5), terminal	Ornamented with short domed spines, 16.8–19.8 (av. 18.3), terminal
Antheridia	Mostly monoclinous, rarely diclinous	Mostly monoclinous, rarely diclinous	Frequently absent, if present obscurely hypogynous or monoclinous	Almost monoclinous	Always monoclinous, rarely diclinous,
Oospores (μm)	Aplerotic, 19.5–26.5 (av. 23.5)	Aplerotic, 16–26 (av. 21.2)	Aplerotic or plerotic, 23–37 (av. 30)	Nearly plerotic, 18.7–25.7 (av. 20.7)	Nearly plerotic, 14.3–17.6 (av. 16.4)
Oospore wall thickness (μm)	1.0–2.5	1.1–2.7	Moderately thick walls	Unknown	Unknown
Cardinal temperature	Min 4 °C, optimum 25 °C and max 35 °C on PCA	Min unknown, optimum 25 °C and max 35 °C on PCA	Min unknown, optimum 20–25 °C and max 32 °C on HSA (extract from 20 g of cracked hempseed in 1 liter of tap-water with 2% agar)	Min 3 °C, optimum 23–25 °C and max 30 °C on CMA	Min 3 °C, optimum 20 °C, and max 30 °C on CMA
Daily growth rates	25 on PCA at 25 °C	22.2 on PCA at 25 °C	15 on HSA at 22–24 °C	19 on CMA at 25 °C	22 on CMA at 25 °C
Reference	This study	Senda et al. (2009)	Hendrix and Campbell (1971)	Otsubo et al. (2025)	Otsubo et al. (2025)

**Table 3:** Morphological description of *Globisporangium monoclinum* and the most closely related species.

Character	*G. monoclinum* (Chen 1725)	*G. monoclinum* (IRAN 2386 C, ex-type strain)	*G. iwayamai*	*G. pengfuense*	*G. violae*
Width of hyphae (μm)	Up to 6	Up to 5	Up to 6.6	Up to 5	Up to 6
Sporangia	Not observed	Not observed	Globose, ellipsoidal, ovoid or limoniform, terminal	Globose to sub-globose, catenulate, terminal, occasionally with apical papillae or intercalary	Not observed
Hyphal swellings (hyphal bodies)	Globose to sub-globose, sometimes filamentous inflated, terminal or intercalary	Globose, occasionally subglobose, terminal or intercalary	Globose, with a rather thick wall, intercalary	Not observed	Terminal and intercalary
Oogonia (μm)	18.8–25.8 (av. 21.5), mostly intercalary, occasionally terminal	16–26 (av. 20.94), mostly terminal, occasionally subterminal or intercalary, rarely in chain	23–29 (av. 27), terminal or intercalary,	12.5–22.5 (av. 17.5), terminal or intercalary	25–38 (av. 29.5), terminal or intercalary
Antheridia	Mostly monoclinous, sometimes hypogynous or diclinous	Mostly monoclinous, rarely diclinous,	Monoclinous or diclinous	Mostly monoclinous, occasionally diclinous	Mostly monoclinous
Oospores (μm)	Plerotic or aplerotic, 15.3–22.6 (av. 18.7)	Plerotic, rarely aplerotic, 10–24 (av. 18.71)	Plerotic or aplerotic, 19–24 (av. 22)	Plerotic or nearly plerotic, 10.5–21.5 (av. 16.5)	Aplerotic, 22–32 (av. 27)
Oospore wall thickness (μm)	1–2	Up to 2	Unknown	0.5–1	Up to 3
Cardinal temperature	Min 4 °C, optimum 25 °C and max 38 °C	Min unknown, optimum 25 °C and max 40 °C	Min 5°C, optimum 25°C and max 30°C	Min 5°C, optimum 25°C and max 35°C	Min 5°C, optimum 25°C and max 35°C
Daily growth rates on PCA at 25 °C	16	14	14	14	15
Reference	This study	Badali et al. (2020)	Van der Plaats-Niterink (1981)	Chen et al. (2022)	Van der Plaats-Niterink (1981)

**Table 4:** Morphological description of *Globisporangium oryzicola* and the most closely related species.

Character	*G. oryzicola* (Chen 509)	*G. oryzicola* (HT2-5, ex-type strain)	*G. kunmingense*	*G. spinosum*	*G. sylvaticum*	*G. terrestris*
Width of hyphae (μm)	Up to 7	Up to 6	Up to 8.6	Up to 5(–7)	Up to 11	Up to 8
Sporangia	Not observed	Not observed	(Sub)globose, ovoid or limoniform, terminal or intercalary	Not observed	Not observed	Globose to somewhat elongated, mostly intercalary
Hyphal swellings (hyphal bodies)	Rarely observed, globose to sub-globose, mainly intercalary, occasionally terminal	Rarely observed, globose to sub-globose, terminal	Not observed	Globose or limoniform, terminal or intercalary	Globose or limoniform, intercalary or terminal	Not observed
Oogonia (μm)	16.5–23.5 (av. 20.4), terminal or intercalary	16.6–24.7 (av. 20.5), terminal, occasionally intercalary	15–26 (av. 21), terminal or intercalary	17–21 (av. 18.5), intercalary, sometimes terminal	18–20 (av. 19.3),terminal or intercalary	18–30 (av. 23.8), mostly intercalary, rarely terminal
Oogonium ornamentation	Absent	Absent	Ornamentations papilliform	With a varying number of blunt, digitate ornamentations	Absent	Absent
Antheridia	Monoclinous or diclinous, sometimes hypogynous	Diclinous or monoclinous	Mostly monoclinous, occasionally diclinous	Monoclinous, occasionally diclinous	Diclinous	Hypogynous or monoclinous
Oospores (μm)	Aplerotic, occasionally plerotic 14.7–19.3 (av. 16.2)	Aplerotic, 13–18.9 (av. 15.8)	Plerotic, 10–24 (av. 19)	Plerotic, occasionally aplerotic, 15–19 (av. 17.2)	Aplerotic, 15–18 (av. 16.5)	Aplerotic, 10–25 (av. 19.9)
Oospore wall thickness (μm)	0.8–1.1	Up to 1	0.8–2	Unknown	1–2	2–4
Cardinal temperature	Min 5 °C, optimum 28 °C and max 35 °C	Min 7 °C, optimum 25–28 °C and max 32 °C	Unknown	Min 5 °C, optimum 25 °C and max 35 °C	Min 5 °C, optimum 25 °C and max 35–40 °C	Unknown
Daily growth rates on PCA at 25 °C	25/per day	26.5/per day	Unknown	30–35/per day	30/per day	9/per day
Reference	This study	Uzuhashi et al. (2017)	Van der Plaats-Niterink (1981)	Van der Plaats-Niterink (1981)	Van der Plaats-Niterink (1981)	Paul (2002)

Phylogenetically both isolates (Chen 1002, 1114) clustered with the ex-type strain of *E. senticosum* according to the ITS and *Cox1*-based phylogeny with high support (99% MP and 1.00 BPPs; **Figure 1**). Moreover, Chinese isolates of *E. senticosum* show high morphological consistency with the original description of *E. senticosum* from Japan (Senda et al., 2009), sharing the ovoid to ellipsoidal sporangia with conspicuous apical papilla, ornamented oogonia with acute spines, mostly monoclinous antheridia, semicircle, subclavate or fist-shaped antheridial cells, and aplerotic oospores. *E. senticosum* from China resembles *E. verrucosum* Otsubo, Kageyama & Hieno, in having mostly semicircular antheridia, but the former species is distinguished in its bigger oogonia (19.5–26.5µm), aplerotic oospores, and slower growth (**Table 2**; Otsubo et al., 2025).

The Chinese isolate of *G. monoclinum* (Chen 1725) forms a lineage with the authorized sequences of *G. monoclinum* IRAN 2421 C from Iran (100% MP and 1.00 BPPs). *G. monoclinum* from China is characterized by globose to sub-globose, sometimes filamentous inflated hyphal swellings, smooth oogonia, mostly monoclinous antheridia, and subcircular, subclavate or fist-shaped antheridial cells. The morphological characteristics of Chinese *G. monoclinum* are similar to the type strain of *G. monoclinum* (**Table 3**, Badali et al., 2020), but the former strain is distinguished in its presence of hypogynous antheridia.

Phylogenetically, all three isolates (Chen 509–511) formed a strongly supported monophyletic group with the ex-type strain of *G. oryzicola* (100% MP and 1.00 BPPs). *G. oryzicola* differs from other *Globisporangium* species by globose to sub-globose, mainly intercalary, occasionally terminal hyphal swellings, subcircular, sub-globose, fist-shaped, subclavate or oval antheridial cells, and thin-walled oospores (0.8–1.1 µm). The Chinese isolates (Chen 509–511) are nearly morphologically identical to the ex-type strain HT2–5 from Japan, but Chinese *G. oryzicola* differ from the typical descriptions of *G. oryzicola* by having hypogynous antheridia and occasionally plerotic oospores (Uzuhashi et al., 2017). Morphologically, both *G. sylvaticum* (W.A. Campb. & F.F. Hendrix) Uzuhashi, Tojo & Kakish., and *G. terrestris* (B. Paul) Uzuhashi, Tojo & Kakish., resemble *G. oryzicola* in lacking oogonium ornamentation. The combined ITS and *Cox1* sequence data support a close phylogenetic relationship among *G. sylvaticum*, *G. terrestris*, and the Chinese isolates of *G. oryzicola* (**Figure 1**). However, *G. sylvaticum* lack monoclinous and hypogynous antheridia and have slightly thicker-walled oospore wall (1–2 µm), while *G. terrestris* is distinguished from Chinese *G. oryzicola* by producing thicker oospore wall (2–4 µm) and slow growth rate (9 vs. 25 mm/day) (**Table 4**; van der Plaäts-Niterink, 1981).

Species of *Elongisporangium* and *Globisporangium* include many economically important plant pathogens that cause damping-off, root rot, and other diseases on agricultural crops, turfgrasses, forest seedlings, and ornamental plants (Uzuhashi et al., 2010; Nguyen et al., 2022). Accurate species identification is therefore critical for disease diagnosis, epidemiological monitoring, and targeted management. The findings of this study substantially advance our understanding of the species diversity and distribution of these two oomycete genera in China.

*E. senticosum* has been documented in association with root decline of *Quercus mongolica* and other hardwood species in natural forest ecosystems in Russia (Malysheva et al, 2013), while *G. oryzicola* was reported as a potential rice pathogen in Japan, with varying levels of aggressiveness among the strains (Uzuhashi et al., 2017). In contrast, knowledge about pathogenicity of *G. monoclinum* is still very much limited. Future studies will focus on evaluating the pathogenicity of these three newly recorded species across relevant host species to assess potential disease risks in forest ecosystems, arid desert regions, and rice-producing areas of China. Overall, these new records expand the known geographic distribution of oomycete species and provide valuable baseline data for biodiversity conservation and plant health management in China.

## Data Availability

The datasets presented in this study can be found in online repositories. The names of the repository/repositories and accession number(s) can be found in the article/supplementary material.
